# Enhanced melanoma and non-melanoma skin cancer classification using a hybrid LSTM-CNN model

**DOI:** 10.1038/s41598-025-08954-8

**Published:** 2025-07-10

**Authors:** Sara M. M. Abohashish, Hanan H. Amin, E. I. Elsedimy

**Affiliations:** 1 Department of Information Technology Management, Faculty of Management Technology and Information Systems, Port Said University, Port Said, Egypt; 2 Department of Information Technology, Faculty of Computers and Artificial Intelligence, Sohag University, Sohag, Egypt; 3 Cyber Secuirty Department, Faculty of Science, Al-Esraa University College, Baghdad, Iraq

**Keywords:** Skin cancer, Long short-term memory, Convolutional neural networks, HAM10000 dataset, Cancer, Diseases, Mathematics and computing

## Abstract

Melanoma is the most dangerous type of skin cancer. Although it accounts for only about 1% of all skin cancer cases, it is responsible for the majority of skin cancer-related deaths. Early detection and accurate diagnosis are crucial for improving the prognosis and survival rates of patients with melanoma. This paper presents a novel approach for the automatic identification of cutaneous lesions by integrating convolutional neural networks (CNNs) with long short-term memory (LSTM) networks. In the proposed approach, the image of each skin lesion is divided into a sequence of tags of a particular size, which is then treated by the LSTM network to capture temporal dependence and relevant relationships between different spatial regions. This patching sequence allows the modeling system to analyze the local pattern in the image. Time CNN layers are later used to extract spatial functions, such as texture, edges, and color variation, on each patch. A Softmax layer is then used for classification, providing a probability distribution over the possible classes. We use the HAM10000 dataset, which contains 10,015 skin lesion images. Experimental results demonstrate that the proposed method outperforms recent models in several metrics, including accuracy, recall, precision, F1 score, and ROC curve performance.

## Introduction

In recent years, skin cancer has become a prevalent form of cancer diagnosed in humans, presenting in various forms such as melanoma and non-melanoma. Over the last few decades, the incidence of both non-melanoma and melanoma skin cancers has increased significantly, with approximately 123,000 melanoma cases and 300,000 non-melanoma cases reported annually^1^. Based on data provided by the World Health Organization (WHO), there are several thousand instances globally that pose a significant risk of mortality by the year 2020. Exposure to harmful UV light is associated with approximately 90% of non-melanoma skin cancers and 86% of melanomas^2^. We categorize skin cancer into two principal types: non-melanoma and melanoma. Cutaneous squamous cell carcinoma (SCC) and Basal Cell Carcinoma (BCC) are two subtypes of non-melanoma skin cancer. Dermoscopy is a non-invasive skin scanning technique that minimizes the reflection of light from the skin’s surface, allowing for the acquisition of detailed and enlarged images of skin lesions to enhance the visibility of spots.

Nevertheless, dermatologists have achieved an accuracy rate of approximately 65–80% in detecting melanoma from dermoscopic images^3,4^. Despite the benign nature of brown spots, small moles, or skin surface rashes, they should not be disregarded. Application of the ABCDE rule, the first skin examination, is necessary to detect any indications of skin lesions that may be progressing into melanoma^5^. The ABCDE rule encompasses visual characteristics such as color, shape, texture, and diameter, which have been utilized in several studies to extract features of skin lesions^6–8^. Early detection and screening for skin cancer may reduce the mortality rate associated with this disease. Initially, skin cancer is often invisible to the human eye because it may appear as a small mole. Furthermore, clinicians face significant challenges in identifying skin cancer disease due to its striking resemblance to other conditions. Moreover, the similarities between skin cancer diseases and other conditions pose significant challenges for doctors during the diagnosis process. Due to numerous constraints in manual skin cancer identification, scientists have turned to artificial intelligence approaches, such as machine learning and deep learning methodologies, for diagnosing the disease. Several studies have employed machine learning models such as K-nearest neighbor (KNN)^9^, decision tree (DT)^10^, and support vector machine (SVM)^11,12^ to enhance the efficiency of automated diagnostic tools. However, these machine learning models do not yield significant results owing to the diverse sizes, shapes, and colors of moles. Furthermore, manual feature extraction is required, a laborious task that demands additional human effort.

Deep learning models have recently shown promise in classifying and recognizing medical images^13^. Many studies have focused on deep learning-based computer-aided diagnosis (CAD) systems^14–16^. Within CAD systems, we can divide deep learning into three processes: preprocessing and parameter setup, deep feature extraction, and diagnosis. Deep learning techniques enable the rapid construction of extensive, low-to-high-level, deep hierarchical feature maps using the same original skin image. Moreover, skin cancer classification employs an advanced deep learning-based approach^17^, overcoming the limitations of traditional machine learning models. There are several models derived from deep learning, such as convolutional neural networks (CNN), recurrent neural networks (RNN), classic neural networks, long short-term memory networks (LSTMs), and others. Researchers are interested in CNN’s potential, and various studies have utilized it for lesion detection^18^, classification^19,20^, segmentation^21^, and image reconstruction^22^ in skin cancer applications. CNNs utilize a variety of building blocks, including convolution, pooling, and fully connected layers, to automatically and adaptively learn feature spatial hierarchies through backpropagation^23,24^. In addition, CNNs enable the extraction of nonlinear features from images, which is crucial for various tasks, such as object detection, semantic segmentation, and image segmentation. CNN’s most efficient task involves classifying and recognizing images, enabling high accuracy through the extraction of feature structures. The MobileNet V2 and LSTM-based deep learning approaches are also effective for skin lesion classification^25^. Mahum et al.^26^ proposed a robust skin cancer detection model based on feature fusion using LSTM. Integrating diverse imaging modalities can achieve a comprehensive evaluation of cancer detection. However, due to the complexity of skin lesion photos, evaluating and analyzing them requires more time and is prone to error. Recent research has utilized a combination of deep neural networks, including LSTM and CNN, to enhance the early detection of skin cancer from dermatological images^27,28^. Kumar et al.^29^ investigated a hybrid approach to detect skin lesions by combining CNN and RNN. To accomplish this, the authors used the HAM10000 dataset. Furthermore, the results confirm that the performance produced a maximum classification accuracy of 94% and 97.3% for CNN and RNN, respectively. A significant challenge in creating a successful automatic classification system is the lack of substantial datasets. The presence of data imbalance and overfitting compromises accuracy. Moreover, researchers suggest integrating two or more machine learning and deep learning algorithms, such as CNN and SVM^30^, CNN and KNN^31^, CNN and random forest (RF)^32^, and CNN and k-means^33^, to enhance accuracy.

Specifically, existing models often struggle with achieving a balanced performance across precision, recall, and overall tracking robustness, particularly in complex or noisy environments. The proposed approach addresses this gap by integrating CNNs and LSTMs to learn spatial and temporal features in images of skin lesions. Through this, the accuracy in detecting melanoma is improved, resolving the limitations of previous models in their inability to learn complex feature patterns effectively. It declares that a new approach, combining CNNs and LSTMs, is proposed, indicating that earlier studies were unable to effectively leverage both spatial and temporal patterns simultaneously for skin cancer detection. Therefore, the use of LSTMs on sequence features is relatively uncommon in image-based medical diagnostic tasks, suggesting that authors aim to address a shortcoming in modeling feature dependency across sequences, perhaps due to preprocessed or enriched data.

### Motivation and contribution

Deep learning plays a significant role in accurately detecting and classifying skin cancer, especially when working with large medical image datasets. CNNs are particularly effective, as they can autonomously extract and learn relevant features from raw data, eliminating the need for manual feature engineering. However, due to the inherently black-box nature of CNNs, Explainable AI (XAI) techniques have been developed to interpret and visualize their internal decision-making processes^34^. These techniques analyze how the model behaves and generate intuitive outputs, such as heatmaps and feature attribution scores, which help users understand which features had the greatest influence on a given prediction. Taghreed et al.^35^ integrated CNN and LSTM in a hybrid model designed to enhance the ability to focus on relevant temporal sequences and spatial features for predicting bladder cancer recurrence and treatment response. The hybrid model was employed to enhance the model’s ability to focus on relevant temporal sequences and spatial features. CNN is applied in^36^ to identify mammographic features, including spatial structures and patterns indicative of malignancy, whereas the LSTM networks capture sequential relationships and temporal dynamics. Despite the numerous approaches proposed for skin cancer detection, there remains a need for an integrated framework that can perform both feature extraction and classification, especially when handling large datasets. Furthermore, the proposed method should deliver more informative and reliable evaluation results, particularly by accounting for both the prevalence of positive and negative samples within the dataset. Furthermore, this paper proposes a novel hybrid deep learning model that combines a long short-term LSTM followed by a CNN for skin cancer classification using the HAM10000 dataset. The LSTM is initially used to capture temporal dependencies from the input data, generating feature sequences that represent important time-based patterns. These features are then passed to the CNN, which extracts spatial features and performs classification. Time-distributed convolutional and pooling layers enable the CNN to apply convolution and pooling operations individually to each temporal segment or frame produced by the LSTM, processing them separately at every time step. This architecture effectively captures both temporal and spatial information for improved classification performance.

The key contributions of this paper can be summarized as follows:

This paper presents a novel hybrid deep learning model that applies LSTM layers before CNN layers to improve the classification of melanoma and non-melanoma skin cancers using the HAM10000 dataset.

- The LSTM component, which was initially to capture temporal dependencies and sequential relationships in the input data, effectively modeling the progression or spatial distribution of skin lesion features.
- The extracted feature sequences are then passed through the CNN component, which performs spatial feature extraction by identifying key visual patterns such as texture, edges, and color variations.
- Time-distributed convolutional and pooling layers enable the CNN to process each temporal segment independently, enhancing the model’s capacity to handle complex and diverse skin lesion characteristics.
- The hybrid model can handle the diversity and complexity of skin lesions more effectively than models that only use CNN or LSTM.
- Experimental results show that the LSTM-CNN hybrid model outperforms traditional architectures in classification accuracy and reliability. This approach supports early and accurate diagnosis of skin cancer, which is especially critical for melanoma, where timely detection significantly increases survival rates.

## Related work

Traditional deep learning-based techniques were introduced for melanoma detection and classification, as presented in Table 1. Ballerini et al.^9^ introduce a new hierarchical classification method that utilizes kernel neural network (KNN) to extract color and texture information from skin lesion images. A hierarchical KNN classifier (HKNN) consists of three separate KNN classifier systems, one positioned at the highest level and two at the lowest level. The highest-level classifier takes input from every image in the training set and systematically classifies them into two distinct groups. The authors exclusively train the remaining two classifiers using images from the respective groups, such as Actinic Keratosis (AK), BCC, and SCC. Viknesh et al.^11^ conducted research on SVM to develop a more effective framework using image processing tools, resulting in an accuracy of 95% based on the dataset provided by the International Skin Imaging Collaboration (ISIC). The gray level co-occurrence matrix (GLCM) method extracted features, which SVM then used to detect skin lesions. The model consists of three phases. It commences with data collection, followed by data augmentation. Finally, the authors present the design of the proposed model. Oliveira et al.^12^ also proposed a hybrid approach to detecting skin cancer that utilizes both SVM and LSTM. It uses the DermIS dataset for feature extraction and classification. The authors manually extracted the features using local binary patterns (LBP) and used Inception V3 for automatic feature extraction. The proposed methodology achieved an accuracy of 99.4%, a precision of 98.7%, a recall of 98.66%, and an F-score of 98%.

**Table 1:** The state-of-the-art techniques for detection skin cancer.

Refs.	Dataset	Model	Activation function	Accuracy	Classes of skin lesion
^10^	ISIC	CNN + SVM	ReLu	91%	Benign, malignant
^19^	PH2, DermIS	CNN	Sigmoid	94.9%	Melanoma, non-melanoma
^20^	SIC-2019 ISIC-2020	DCNN	ReLu	–	Melanoma, non-melanoma
^21^	7-Point, Med-node, PAD-UFES-20 and PH2	ANN		96.7%	Melanoma, nevus, dysplastic nevus
^26^	DermIS	LSTM	–	99.4%	Melanoma, benign lesions
^27^	ISIC 2017	CNN + LSTM	Sigmoid	94.6%	Melanoma, benign lesions
^28^	18274 dermoscopy images	CNNs and LSTMs	Sigmoid	93.41%	Benign, malignant
^29^	HAM 10000	CNN + RNN		94%	Melanoma, nevi, dermatofibroma, seborrheic keratosis, BCC, and SBC
^30^	ISIC	CNN + SVM Sparse Coding,		93.1%	Melanoma, atypical nevi, benign lesions
^32^	ISIC 2019	DCNN + RF + Naïve Bayesian	Sigmoid	99.5%	Melanoma, solar lentigo, seborrheic keratosis

The authors in ref.^18^ introduced a new model for skin cancer to classify nine categories of clinical types. CNN used the softmax function as an activation function. The authors also examined VGG-16 and VGG-19, achieving accuracies of 69.57% and 71.19%, respectively. Moura et al.^19^ introduced a novel method that integrates the ABCD rule with CNN to assist dermatologists in identifying skin lesions. They selected and trained the features using a multilayer perceptron classifier. The Kappa index was 99.70%, and the accuracy was 94.9%. The authors in ref.^20^ applied different deep CNNs to classify skin cancer. To enhance its accuracy, the EfficientNet design employed transfer learning approaches to provide a pre-trained model. The primary objective of the EfficientNet architecture is to improve accuracy by leveraging the compound scaling technique. In this study, the authors performed experiments utilizing two distinct datasets, specifically ISIC-2019 and ISIC-2020. Each of these datasets exhibited unique image resolutions and class imbalance problems. Using standard digital images, Moldovanu et al.^21^ explored the ABCD characteristics as lesion descriptors to diagnose melanocytic and non-melanocytic lesions. They conducted this study to compare and contrast the two types of lesions. The authors conducted experiments on four different datasets comprising dermoscopic and non-dermoscopic images.

Meanwhile, Naqvi et al.^23^ focused on developing a deep CNN technology for skin cancer prediction. They used a GoogleNet model to diagnose skin cancer, using photos of the disease as inputs. They implemented techniques such as convolution, pooling, softmax, and fully linked processes, achieving an accuracy range of 75–85%. On the other hand, Sai et al.^24^ utilized gradient descent, the rectified linear unit (ReLU), and various optimizers to extract essential features through self-learning and training models. When compared to conventional models, the proposed method demonstrates superior performance and a higher level of accuracy, reaching 75% or higher. Mahum et al.^26^ have developed a new automated process that utilizes handcrafted feature extraction from skin images, conducted in three steps. First, they applied Gaussian filtering (GF) to the image for preprocessing to eliminate noise. Second, we manually performed feature extraction on the DermIS dataset using linear block programming (LBP) and Inception V3 with Adam optimizer. Finally, they employed LSTM to identify and categorize the skin images as either melanoma or benign lesions. Sequentially, Kohli et al.^27^ integrated two deep learning algorithms, CNN and LSTM, for the automated diagnosis of skin lesions. The authors extracted CNNs from images by learning hierarchical features from dermoscopy images and utilized LSTM to model temporal dependencies and understand the hierarchical structure of the data. Authors^28^ have conducted similar research, improving discriminative feature learning by combining CNN-LSTM with the pre-trained InceptionResNetV2 model. The detection of skin cancer achieved high precision and minimal false-positive rates.

Codella et al.^30^ used CNN and SVM to extract characteristics from the ISIC dataset of skin lesion images. The authors conducted experimental trials using a total of 2624 images, which included 334 images of melanoma, 144 images of atypical nevi, and 2146 images of benign lesions. The results indicated that the accuracy attained for the first and second tasks was 93.1% and 73.9%, respectively. Kulkarni et al.^31^ have recently classified skin lesions as either melanoma or non-melanoma, further dividing non-melanoma into BCC and SCC. The authors employed CNN to classify malignant tumors as either melanoma or non-melanoma. The authors employed numerical classifiers, such as KNN and SVM, to categorize non-melanoma lesions into basal cell carcinoma and squamous cell carcinoma. The proposed methods achieved superior accuracy when applied to the existing dataset in comparison to alternative approaches. The authors in ref^32^ presented a novel methodology that combines machine learning and deep learning to identify and diagnose skin cancer. The authors employed a novel deep CNN to extract structural features and trained classifiers based on the color, boundaries, and texture of skin lesions using Naive Bayes and random forest (RF) algorithms. The proposed approach outperforms the existing comparable approaches in terms of accuracy. The Naive Bayes and RF algorithms have achieved accuracy rates of 96% and 97%, respectively. Subsequently, Bharathi et al.^33^ introduced image processing techniques for identifying melanoma from nevus images. The authors employ a median filter to eliminate noise from the input image and then use enhanced K-means clustering to segment it. The authors distinguish melanoma and nevus using the adaptive neuro-fuzzy inference system (ANFIS) and feed-forward neural network (FFNN) in conjunction with the DERMIS dataset. They conducted the investigations using 1023 images, comprising 104 melanoma and 919 nevus images. The accuracy of the ANFIS and FFNN classifiers was 97.3% and 96.8%, respectively, as a result of the proposed model.

In the same context, Ullah et al.^37^ implemented occlusion sensitivity as XAI to identify and visually indicate the image regions most relevant to classification. It utilized DeepCRINet for the recognition of melanoma. On the other hand, Ullah et al.^38^ proposed a lightweight model, ChestCovidNet, for detecting COVID-19 from various chest radiograph image (CRI) datasets. ChestCovidNet is an efficient CNN designed with eight convolutional layers and three fully connected layers. Additionally, it incorporates two normalization techniques cross-channel normalization and batch normalization—to enhance training stability and model performance. DeepLCCNet was developed in^39^ to classify five types of tissues: squamous cell lung cancer, colon cancer, benign colon tissue, benign lung tissue, and lung cancer. Meanwhile, the extraction of high-value features was implemented in^40^ by integrating a CNN with a Vision Transformer. Rehman Khan et al.^41^ improved deep learning performance by employing multiple imaging modalities. Many studies have demonstrated the effectiveness of these techniques in various medical imaging applications^42–47^.

## Proposed model

**Fig. 1:**
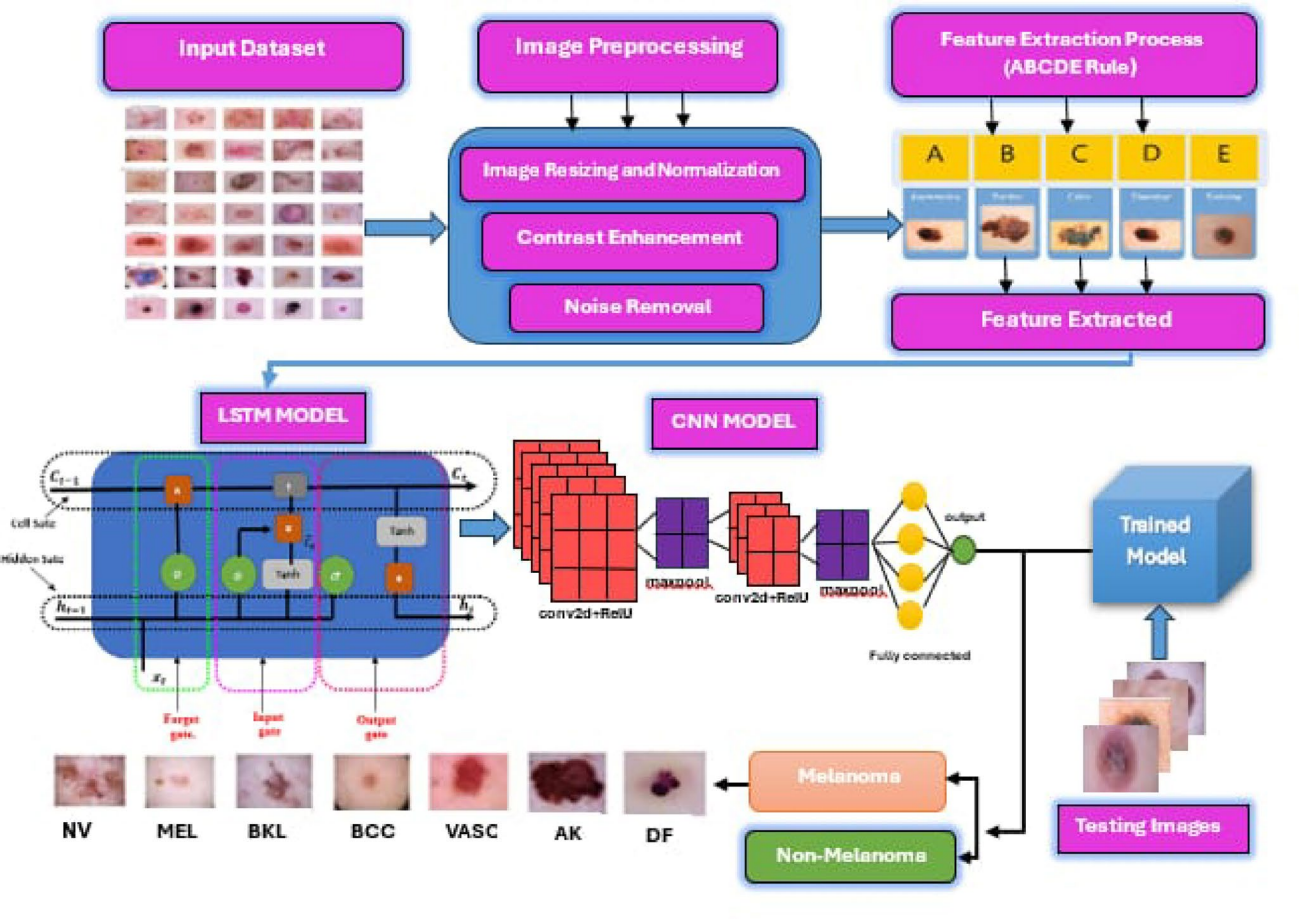
The hybrid LSTM-CNN model for skin cancer detection.

This work proposes a skin cancer detection method based on LSTM-CNN. The model consists of three sequential stages, as depicted in Fig. 1. Initially, the procedure involves constructing a sequential model, preprocessing the image data, specifying the model architecture, compiling the model, and then training it using the skin cancer dataset. Then, it establishes an LSTM layer to extract temporal dependencies from the sequence of feature vectors. The LSTM configuration has input, output, forget, and cell gates. The cell functions as the memory component of the LSTM device. Each of these gates plays a distinct role in the LSTM’s operation. Subsequently, the CNN layers utilized time-distributed layers to apply convolutional and pooling operations to each frame individually. The CNN model independently subjects each temporal slice of an input or each frame (or time step) to the same layer. The softmax layer generates the probability distribution across the different classes in classification tasks. A max-pooling layer maximizes the spatial dimensions (height, breadth) of the feature maps. The dense layers, derived from the output of the CNN layer, achieve the final classification of the hybrid LSTM-CNN model.

### Data preprocessing

The extraction of features from skin images is a crucial process in various dermatological and cosmetic applications, enabling the analysis of skin texture and the identification of abnormalities. Feature vectorization is the process of converting extensive input data into a low-dimensional representation, thereby reducing the resources required to describe a large dataset. The ABCDE rule is a widely used technique for skin cancer detection^7^. The chosen parameters include shape properties such as asymmetry and border irregularity, color variation, diameter, object area, and roundness. The extracted features can be combined into a feature vector x for further analysis as cluster X = [A, B, C, D]. The proposed model may use this vector as input to categorize skin cancer lesions as benign or malignant. The ABCDE rule stands for:

#### Shape

The shape exhibits two-dimensional asymmetry and uneven borders. The asymmetry feature contains information about the lesion’s asymmetry and lengthening index. Initially, we calculate it by dividing the lesion’s segmented area into two equal parts and then comparing them. Then, we classify the lesion as symmetric when the two sub-regions show a high degree of similarity; otherwise, we regard it as asymmetric. Subsequently, we determined the lesion asymmetry index by computing the combined area of the lesion’s inner and outer regions.

1 $$AI = \frac{\Delta AK}{{AL}}$$

where *AL* is the lesion area and Δ*AK* is the difference between the two halves of the lesion and can be calculated as follow:

2 $$AK = \frac{1}{2}\, {{}_{i = 1}\!\!\!{}^{n}}\!\! \left| {f\left( {x_{i} ,y_{i} } \right) - f\left( {x_{i}^{\prime} ,y_{i}^{\prime} } \right)} \right|$$

where ${ }f\left( {x_{i} ,y_{i} } \right)$ represents the pixel intensity or color at point $\left( {{\text{x}},{\text{y}}} \right)$ and $\left( {x_{i}^{\prime} ,y_{i}^{\prime} } \right)$ are the corresponding points on the opposite half of the lesion.

The measurement of a lesion’s border determines its irregularity. In most cases, a benign lesion will have a border that is regular and even in shape, but a border that is uneven in shape is frequently symptomatic of a border that is malignant. By comparing the real perimeter to a smooth or convex hull perimeter, one can determine the degree of irregularity that exists along the boundary.

3 $${\text{B}} = \frac{{\text{Actual perimeter}}}{{\text{Convex hull perimeter}}} = \frac{{P_{L}^{2} }}{4\pi AL}$$

where $P_{L}$ is the perimeter lesion.

#### Color

The process involves assessing the color variegation within pigmented lesions. Malignant melanoma lesions typically present with non-uniform colors, which can be an important diagnostic feature. Additionally, we can measure the color variation by calculating the standard deviation as follows:

4 $${\text{C}} = {\text{std}}\left( {\left\{ {c_{i} } \right\}} \right)$$

where $c_{i}$ represents the color value at pixel *i*.

#### Diameter

The diameter criterion refers to the lesion’s size. While many benign moles are small and remain the same size over time, melanomas often grow larger. Lesions exceeding 6 mm in diameter are more suspicious and warrant closer examination. Diameter is evaluated as follows:

5 $${\text{D}} = \sqrt {\frac{4*AL}{\pi }}$$

#### Roundness

The roundness refers to a geometric measure of how closely a lesion resembles a perfect circle. The circularity ratio is a common way to measure roundness. To determine the lesion area, divide its area by the area of a circle with the same circumference as the lesion.

6 $${\text{Roundness }} = \sqrt {\frac{4\pi *AL}{{P_{L}^{2} }}}$$

where *AL* is the area of the lesion, and perimeter is the total length of the lesion’s boundary. A perfectly round lesion would have a circularity ratio close to 1. As the object becomes more irregular in shape, the ratio decreases.

#### Area

The area of lesion refers to the measurement of the two-dimensional space or surface occupied by lesion. That can be accomplished by changing the image to monochromatic and then segmenting the lesion.

### LSTM

#### Learning spatial and temporal features

LSTM is a specialized form of RNN structure designed to address the issue of the disappearing gradient in conventional RNNs and effectively capture extended dependencies within sequential data. The LSTM architecture consists of input, output, forget, and cell gates. The cell is the memory part of the LSTM. Each of these elements plays a distinct role in how the LSTM operates^48^.

**Fig. 2:**
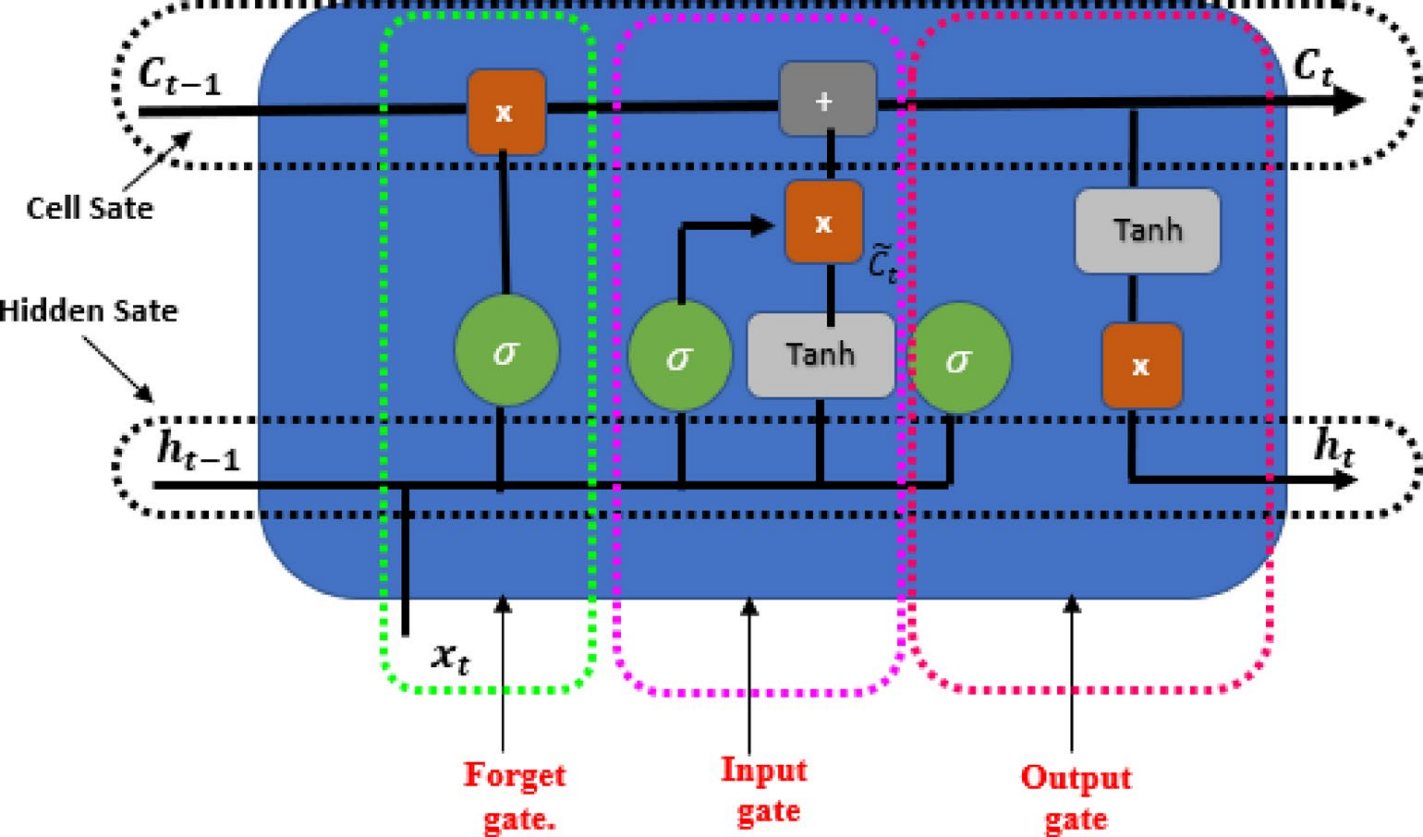
LSTM memory cell.

A cell can store or forget specific information because it retains the state of a sequence in its memory. The forget gate determines whether to maintain or erase data, the input gate controls the amount of data stored, and the output gate determines the subsequent state^49^. Here, a series of images (w_1_, w_2_, w_3_, …, w_n_) are processed one by one. At time step t, h_t_ and C_t_ represent the hidden state and the cell state of the LSTM, respectively, as shown in Fig. 2.

**Fig. 3:**
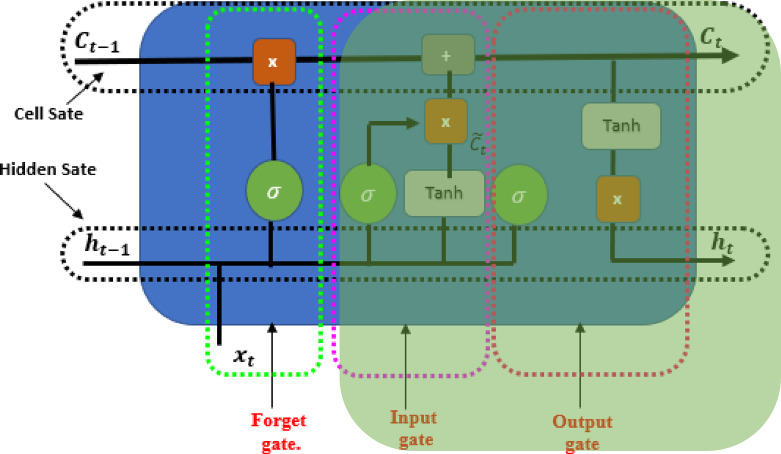
Forget gate of LSTM.

Additionally, the value of the forget gate is employed to decide the proportion of the prior cell state $C_{t - 1}$ that needs to be kept or discarded as shown in Fig. 3. The forget gate is calculated using the sigmoid activation function as follow:

7 $$f_{t} = \sigma \left( {W_{f} \cdot \left[ {h_{t - 1} ,x_{t} } \right] + b_{f} } \right.$$

where $W_{f}$ and $b_{f}$ are a collection of parameters of sigmoid activation function.

**Fig. 4:**
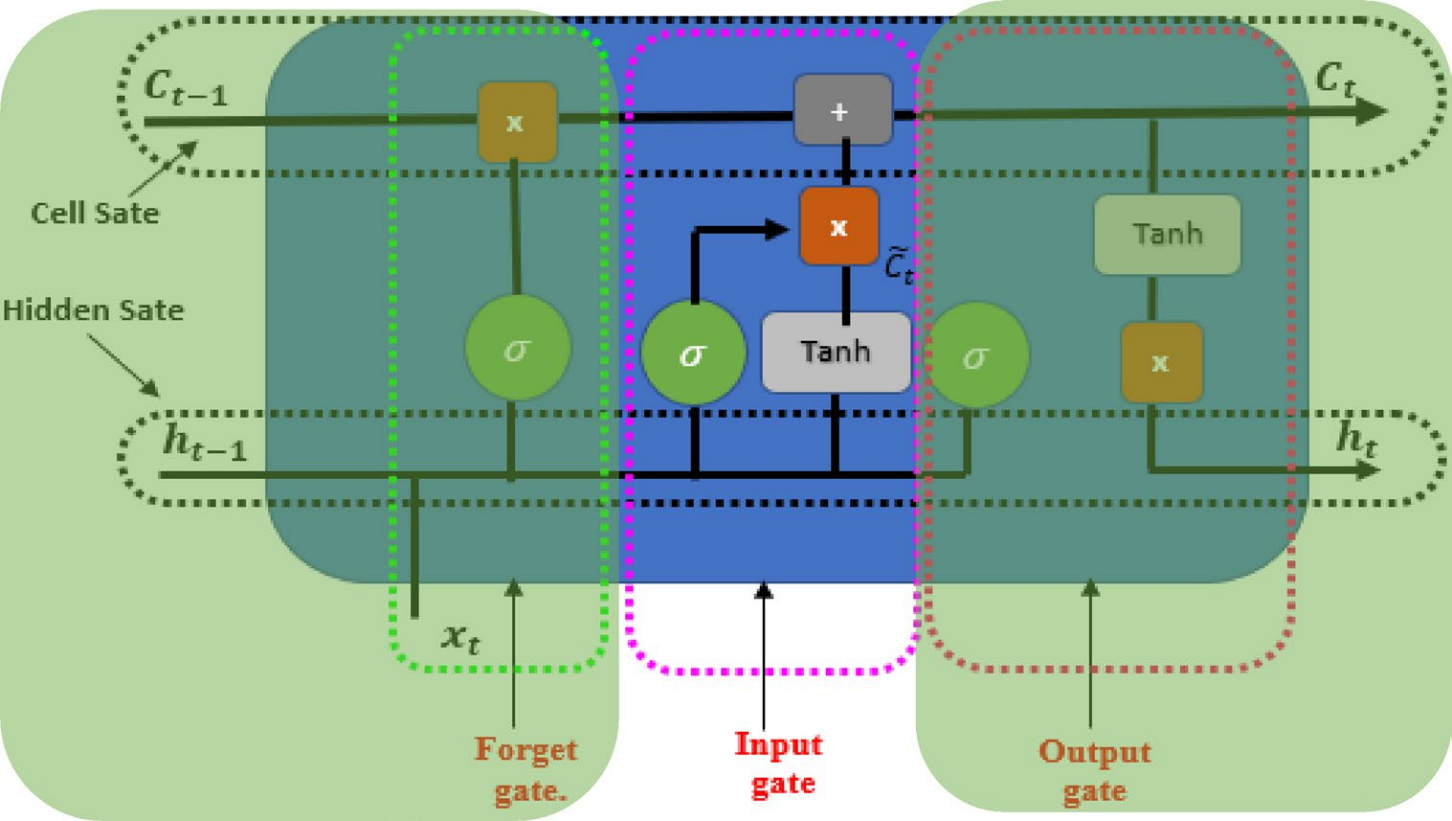
Input gate of LSTM.

After that, as shown in Fig. 4, the input gate should update the cell state with the new candidate values. We utilize the sigmoid function in conjunction with the information door.

8 $$i_{t} = \sigma \left( {W_{i} \cdot \left[ {h_{t - 1} ,x_{t} } \right] + b_{i} } \right.$$

Next, the input gate of LSTM updates candidate values for the cell state by applying the hyperbolic tangent activation, as follows:

9 $$\widetilde{{C_{t} }} = {\text{tanh}}\left( {W_{c} \cdot \left[ {h_{t - 1} ,x_{t} } \right] + b_{c} } \right.$$

The cell state ($C_{t}$) is improved by merging the previous cell state with the new candidate values, according to the forget and input gates as follows:

10 $$C_{t} = f_{t} *C_{t - 1} + i_{t} *\widetilde{{C_{t} }}$$

**Fig. 5:**
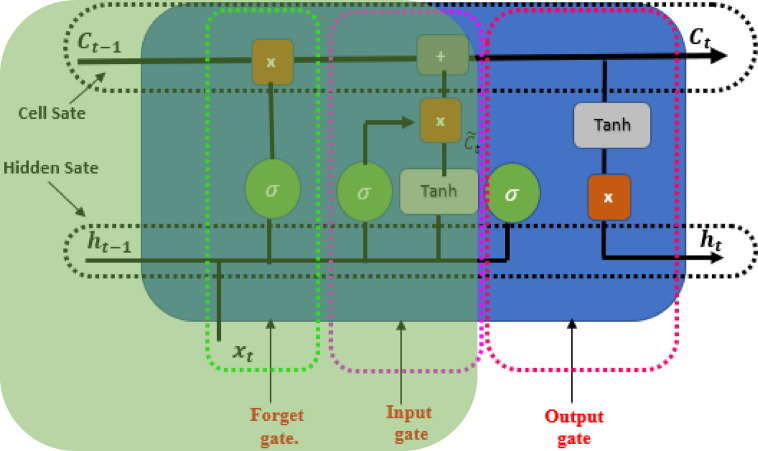
Output gate of LSTM.

Finally, the output gate is calculated by using the sigmoid activation function along with the parameters $W_{0}$ and $b_{0}$ to determine the amount of the cell state that should be shown as the output in Fig. 5.

11 $$O_{t} = \sigma \left( {W_{o} \cdot \left[ {h_{t - 1} ,x_{t} } \right] + b_{o} } \right)$$

The output gate is applied to the updated cell state’s hyperbolic tangent in order to calculate the hidden state as follows:

12 $$h_{t} = o_{t} *\tanh \left( {C_{t} } \right)$$

#### Patch-wise sequence

In computer vision, especially in *Vision Transformers (ViTs)*, images are often processed as *patch-wise sequences*. Given an input $X \in {\mathbb{R}}^{H \times W \times C}$, H, W,C represent the height, the width of image, and number of channels (e.g., 3 for RGB images), respectively.

The image is divided into non-overlapping patches of size P × P. The total number of patches is calculated as:

13 $$N = \frac{H \times W}{{P^{2} }}$$

Each patch is then flattened into a vector of size $P^{2} \cdot C$, and these vectors are projected into a lower-dimensional embedding space using a learnable linear transformation:

14 $$z_{i} = E \cdot x_{i} \quad for\;i = 1,2,3, \ldots N$$

$x_{i} \in {\mathbb{R}}^{{P^{2} .C}}$ is the flattened patch, $E \in {\mathbb{R}}^{{\left( {P^{2} .C} \right) \times D}}$ is the embedding matrix, $z_{i} \in {\mathbb{R}}^{D}$ is the resulting embedded patch vector.

These embedded patch vectors form the input sequence to the Transformer model, often with a special classification token^50^ added at the beginning and positional embeddings $E_{pos}$ added to retain spatial information:

15 $$Z_{0} = \left[ {z_{CLS} ; z_{1} ;z_{2} ; \ldots z_{N} } \right] + E_{pos}$$

This patch-wise sequence approach reduces the input size and complexity while allowing the model to learn relationships between different parts of the image.

As seen in Algorithm 1, the LSTM algorithm for skin cancer detection can autonomously and flexibly acquire spatial hierarchies of characteristics, making it suitable for skin image processing and assessment. LSTM is a distinct category of RNNs capable of developing long-term dependencies. It specifically engineers them to avoid the phenomenon of long-term reliance. The LSTM models receive the input vector x_t_, the previous cell state C_t_ − 1, and the previous hidden state h_t_ − 1 at each time step. The algorithm comprises multiple sequential stages. Initially, the forget gate determines whether to retain or discard the information from the previous cell state. The input gate then arbitrarily chooses the new data to store in the cell state. We update the cell state by aggregating the prior cell state with the latest candidate cell state, assigning weights based on their individual gate activations. Subsequently, the output gate determines the output based on the cell’s current state. The output gate’s calculation is based on the components of the weight matrix and bias vector. Activating the output gate to adjust the cell’s state determines the hidden state at the current time step. Here, the LSTM model retrieves the hidden state and cell state of the current time step and then advances to the subsequent time step.

**Algorithm 1:**
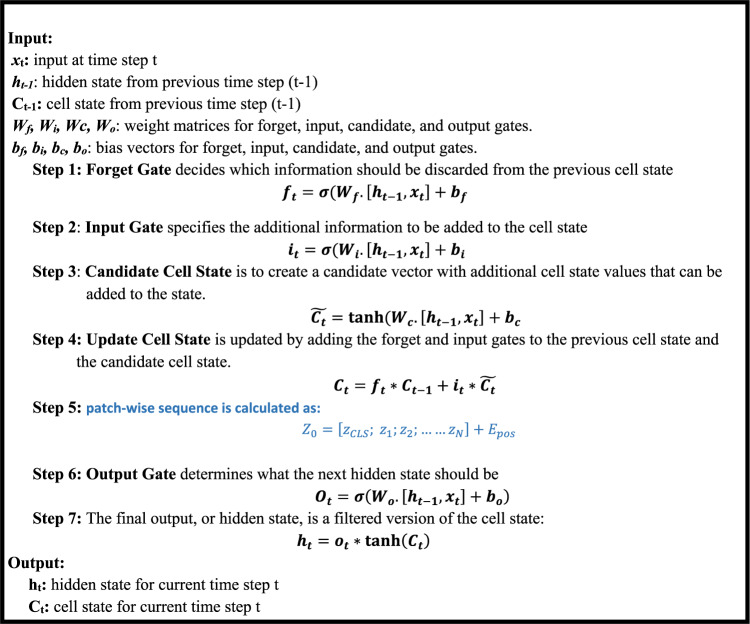
LSTM algorithm.

### Skin cancer classification based on CNNs

CNNs, which fall under the category of deep neural networks, primarily perform visual image analysis. They have achieved remarkable success in various computer vision tasks, including image classification, object identification, and segmentation^18–22^. Additionally, CNNs can automatically and adaptably recognize feature spatial hierarchies based on input data^51^. Their construction includes several layers, including convolutional layers, pooling layers, and fully connected layers. The initial layer corresponds to the convolutional layer, which is responsible for applying a convolutional procedure to the input image^52^. The subsequent section will describe the link between each neuron in the convolutional layer and its corresponding receptive field, which is a relatively small region within the input image.

16 $$C\left[ {i,j} \right] = \mathop \sum \limits_{m} \mathop \sum \limits_{n} I[i + m,j + n] \cdot K\left[ {m,n} \right] + b$$

where $C\left[ {i,j} \right]$ is the output feature map at position $\left( {i,j} \right)$, $I\left[ {i + m,j + n} \right]$, the input data at position $\left( {i + m,j + n} \right)$, $K\left[ {m,n} \right]$ is the convolution kernel (or filter) at position $\left( {m,n} \right)$ and *b* is the bias term.

After the convolution process, the network integrates a non-linear activation function like ReLU to present non-linearity into the CNN network^53^

17 $$f\left( x \right) = {\text{max}}\left( {0,x} \right)$$

**Fig. 6:**
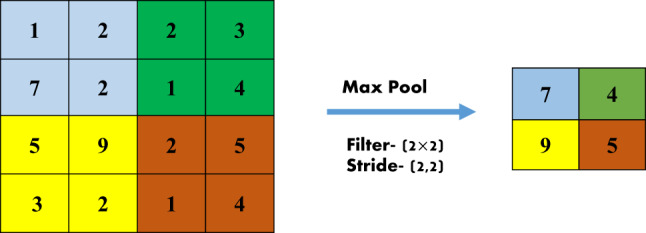
Max pool operation.

As shown in Fig. 6, we also employ the pooling layer to reduce the spatial dimensions of the input volume. We use the max pooling equation as follows to reduce the network’s computational complexity and parameter count:

18 $$O\left[ {i,j} \right] = \mathop {\max }\limits_{m,n} I\left[ {i \times s + k,j \times s + l} \right]$$

where *k* and *l* are pooling dimensions and *s* is the stride (the amount by which the pooling window shifts).

Fully connected layers, following a sequence of pooling and convolutional layers, realize the advanced cognitive processes in the neural network. The above equation establishes a relationship between each neuron in a fully connected layer and every activation in the layer directly below it.

19 $$z = f\left( {Wx + {\text{b}}} \right)$$

where *z* is the output vector, *W* is the weight matrix, *x* is the input vector, *b* is the bias vector and *f* is the activation function^54,55^.

In classification tasks, the softmax layer is used as the output layer to produce a probability distribution over the classes as follow:

20 $$P\left( {y = j{|}X} \right) = \frac{{e^{{Z_{j} }} }}{{\mathop \sum \nolimits_{k = 1}^{k} e^{{Z_{k} }} }}$$

where $P\left( {y = j{|}X} \right)$ is the probability of the jth class, $y_{j}$ is the output of the network for the jth class, and $z_{j}$ is the output of neuron *j*.

The CNN skin cancer algorithm is capable of automatically and adaptively learning spatial feature hierarchies, making it well-suited for processing and evaluating skin images, as shown in Algorithm 2. The initial layer of the model extracts important characteristics from the RGB image input through convolutional procedures. This is followed by the application of a non-linear activation function, such as ReLU. The convolutional neural network also includes pooling layers, like max pooling, which help reduce the spatial dimensions of the convolved features. Additionally, each feature map produced is transformed into a single vector. This vector is then fed into fully connected layers, where each neuron in one layer connects to every neuron in the subsequent layer. Finally, the softmax function is employed for classification to determine whether the patient has non-melanoma or melanoma.

**Algorithm 2:**
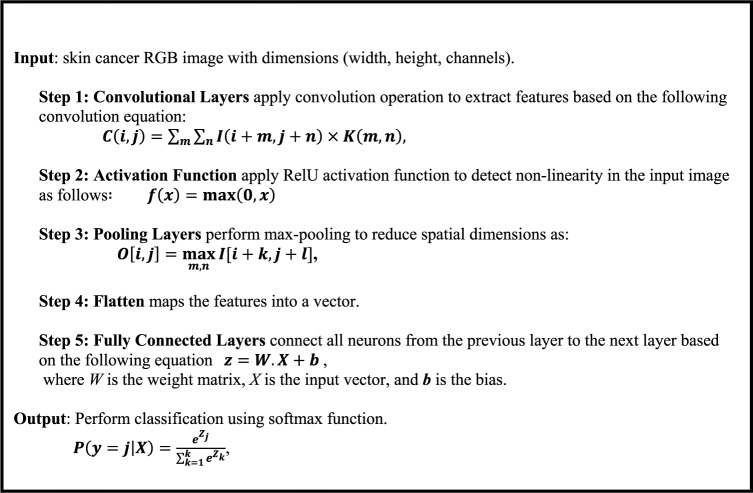
CNN algorithm.

### Hybrid skin cancer detection model

Integrating LSTM networks and CNNs into a hybrid model offers an effective approach for identifying skin cancer. This hybrid model takes advantage of the strengths of both architectures, as illustrated in Algorithm 3. LSTM is a type of recurrent neural network designed to detect and analyze long-term dependencies and sequential patterns in numerical data. This makes it particularly well-suited for analyzing time-series data and sequences. LSTMs regulate the flow of information through input, output, and forget gates, and they include memory cells to retain crucial information over time. On the other hand, CNNs are highly efficient at processing grid data, such as images, by autonomously and flexibly capturing spatial hierarchies of features. These hierarchies encompass a range of characteristics, from fine details to broader patterns. The typical architecture of CNNs includes fully connected layers, pooling layers, and convolutional layers. By combining LSTMs and CNNs, the hybrid model can identify sequential patterns or dependencies among the extracted features. This capability is particularly valuable in situations with a series of images or when incorporating synthetic sequences that illustrate different perspectives or temporal trends of the injury. The hybrid model effectively utilizes the spatial feature extraction abilities of CNNs alongside the sequential pattern recognition strengths of LSTMs, making it well-suited for complex tasks like skin cancer detection through medical image analysis. To prevent overfitting in the hybrid CNN-LSTM architecture, several regularization techniques were employed. Dropout layers with a rate of 0.3–0.5 were added between activation and LSTM layers. Data augmentation techniques—such as rotation, flipping, scaling, and brightness adjustment—were applied to enhance the diversity of the training data. Early stopping was implemented based on validation loss to avoid overtraining. Additionally, L2 regularization with a factor of 0.001 was used to constrain model complexity, and learning rate scheduling was applied to gradually reduce the learning rate.

**Algorithm 3:**
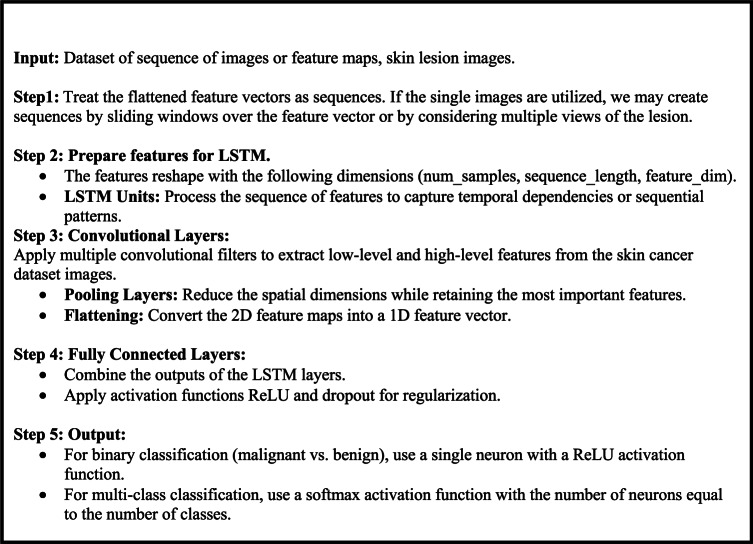
Hybrid LSTM + CNN model for skin cancer detection.

## Experimental results

### Dataset description

To differentiate between melanoma and non-melanoma cases, we evaluated the proposed model using HAM10000^24^, a benchmark dataset for skin cancer that comprises 10,015 images of pigmented skin lesions. This dataset contains six distinct types of irregularities, all of which adversely affect the health of the beehives. The model identifies various types of skin cancer along with their corresponding abbreviations: BCC, pyogenic granulomas and hemorrhage (pyogenic gg), AK, and dermatofibroma. We utilized 80% of the images from the HAM10000 dataset for training purposes and reserved 20% for testing. In accordance with the data, the random state value is set to 42, while the sample fraction value is 0.07. Specifically, the training set includes 985 cases of melanoma, 905 cases of benign keratosis, 430 cases of basal cell carcinoma, 298 cases of actinic keratosis, 101 cases of vascular lesions, and 67 cases of dermatofibroma.

### Environment parameters configuration

**Fig. 7:**
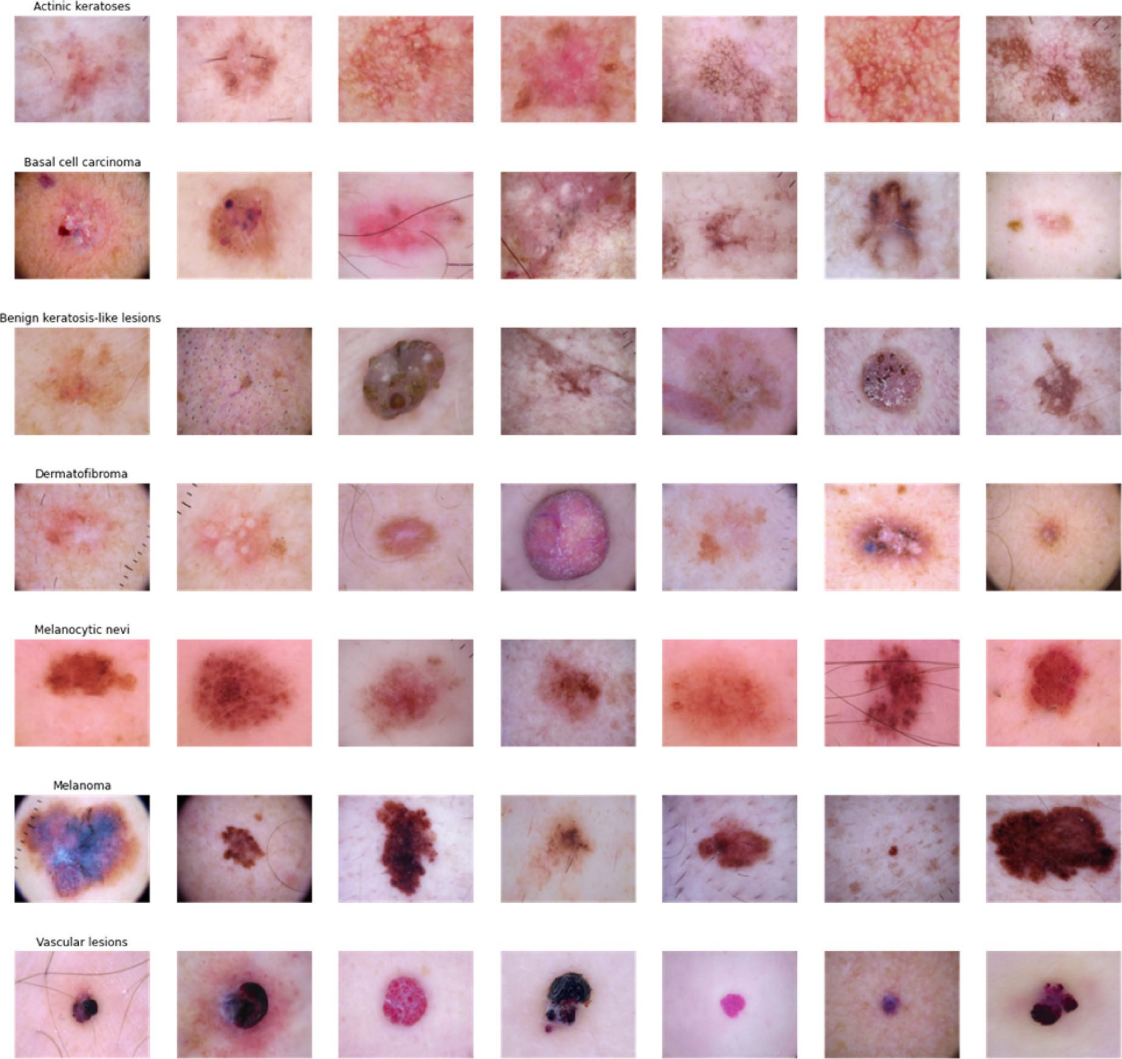
The original skin cancer image with seven classes namely actinic keratosis, basal cell carcinoma, pigmented benign keratosis, dermatofibroma, melanocytic nevi, vascular lesions, and melanoma.

Google Colab, with its GPU, is the environment for learning and optimization. The most often used Python packages are Tensorflow, Keras, OpenCV, Pandas, and Matplotlib. We use random dataset shuffles during the learning process. The photos are reduced to (100 × 100 × 3) and (128 × 128 × 3) for classification and segmentation, respectively, in the RGB color space. Figure 7 illustrates that color images consist of three components: red, green, and blue.

### Comparative analysis of many classifiers

**Fig. 8:**
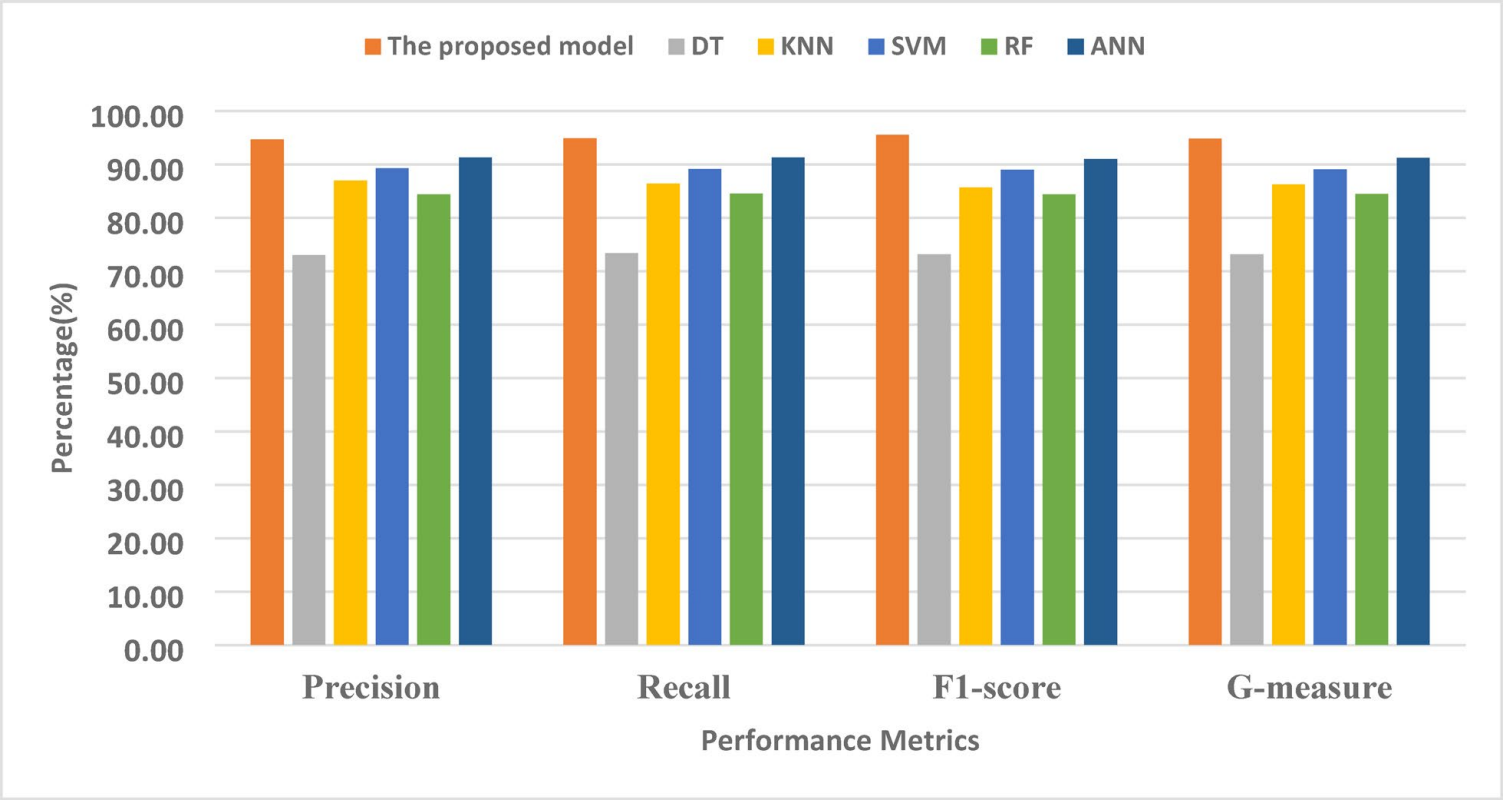
Comparative analysis of the proposed model in terms of precision, recall, F1-score, and G-measure.

This section evaluates all competing classification methods, namely DT, SVM, RF, KNN, ANN, and the proposed model, using the HAM10000 dataset based on the hyperparameter optimization of classification models in Table 2. The purpose is to validate the proposed model’s superiority in detecting and classifying pigmented skin lesions. The results in Table 3 indicate that DT, SVM, RF, KNN, ANN, and the proposed model achieved accuracy rates of 73.67%, 89.23%, 85.89%, 86.56%, 91.78%, and 96.21%, respectively. Furthermore, Table 2 demonstrates that the proposed model yields the best results for precision (94.75%), recall (94.93%), F1-score (95.55%), G-measure (94.81%), and accuracy (96.21%). Furthermore, the DT and RF classification algorithms achieved relative accuracy of 73.67% and 85.89%, respectively. With a precision of 73.94%, a recall of 73.43%, an F-score of 73.14%, and a G-measure of 73.19%, further research indicates that the DT classifier has not performed well. Moreover, RF achieved a higher result with a precision of 84.43%, recall of 84.57%, F1-score of 84.67%, G-measure of 81.1%, and accuracy of 85.89%. In contrast, the KNN classifier achieved a precision of 87.23%, a recall of 86.43%, an F1-score of 85.71%, a G-measure of 86.26%, and an accuracy of 86.56% improvement. The SVM classifier has achieved fair efficiency, with a precision of 89.29%, a recall of 89.14%, an F1-score of 89.33%, a G-measure of 89.11%, and an accuracy of 89.23%. Furthermore, the artificial neural network classifier improved the results, achieving a precision of 91.29%, recall of 90.56%, F1-score of 91.67%, G-measure of 91.24%, and accuracy of 91.78%. In comparison to the current models, the suggested model achieved the highest accuracy (94.75%) and recall (95.56%). Furthermore, Fig. 8 demonstrates that the proposed model surpasses the other models in terms of precision, recall, F1 score, and G-measure.

**Table 2:** The hyperparameter optimization of classification models.

Model classification	Tuned parameters	Tested values	Best values	Optimization method
SVM	Kernel, C, Gamma	C: 0.1–100; Gamma: 0.001–1; Kernels: RBF, Poly	C = 10, Gamma = 0.01, Kernel = RBF	Grid search
KNN	k (neighbors), Distance	k: 3–11; Metrics: Euclidean, Manhattan	k = 5, Euclidean	Manual tuning
RF	n_estimators, Max Depth	Trees: 100–500; Depth: 10–50	Trees: 200; Depth: 30	Random search
CNN	Architecture, LR, Epochs, Batch Size	LR: 0.0001–0.01; Epochs: 50–200; Batch: 16–64	LR = 0.001; Epochs = 100; Batch = 32	Manual and adaptive

**Table 3:** The results of the multi-classification for the HAM10000 dataset.

Models	Precision (%)	Recall (%)	F1-score (%)	G-measure (%)	Accuracy (%)
DT	73.94	73.43	73.14	73.19	73.67
SVM	89.29	89.14	89.33	89.11	89.23
RF	84.43	84.57	84.67	84.16	85.89
KNN	87.23	86.43	85.71	86.26	86.56
ANN	91.29	90.56	91.67	91.24	91.78
The proposed model	94.75	94.93	95.55	94.81	96.21

Furthermore, we conducted tests to evaluate the performance of the classifiers in a multi-class classification scenario. To be more exact, we treated each category of pigmented skin lesions as an independent class. We evaluated the classifiers’ capacity to identify cases of melanoma and non-melanoma, as shown in Table 4. Evidence suggests that the proposed model outperforms competing algorithms across various categories of pigmented skin lesions. In certain scenarios, the proposed model demonstrates a substantial enhancement over ANN and SVM. By comparison, the suggested model achieved a superior F1-score of 92.18% on the melanoma images, surpassing the F1-scores of 80.23% and 85.45% obtained by SVM and ANN, respectively. Furthermore, the scores for basal cell carcinoma images are 96.52%, 92.67%, and 93.78%, respectively. In the most diverse types of pigmented skin lesions, the suggested model consistently achieves an F1 score over 92%.

**Table 4:** The multi-classification results for six classes.

Model	Metric	Precision (%)	Recall (%)	F1-score (%)	G-measure (%)
The proposed model	Actinic keratoses	92.23	94.76	94.47	93.49
	Basal cell carcinoma	96.23	95.78	96.52	96.00
	Benign keratosis-like	95.45	89.12	92.33	92.23
	Dermatofibroma	93.89	95.78	96.69	94.83
	Melanocytic nevi	96.67	96.77	96.79	96.72
	Pyogenic granulomas and hemorrhage	93.56	96.23	99.85	94.89
	Melanoma	94.89	96.09	92.18	95.49
DT	Actinic keratoses	76.45	76.28	76.33	76.45
	Basal cell carcinoma	73.67	77.33	75.89	74.97
	Benign keratosis-like	65.98	63.56	64.13	63.99
	Dermatofibroma	86.08	88.78	87.45	86.99
	Melanocytic nevi	64.15	58.45	61.23	60.93
	Pyogenic granulomas and hemorrhage	86.45	92.13	89.67	88.95
	Melanoma	61.28	60.34	60.18	60.50
KNN	Actinic keratoses	88.23	98.56	94.45	93.91
	Basal cell carcinoma	90.28	92.78	91.16	90.99
	Benign keratosis-like	74.09	83.89	78.34	78.37
	Dermatofibroma	88.13	94.77	93.78	93.81
	Melanocytic nevi	78.72	73.45	72.89	73.97
	Pyogenic granulomas and hemorrhage	89.43	98.45	94.23	94.34
	Melanoma	82.34	75.67	78.57	78.42
SVM	Actinic keratoses	93.45	99.05	96.23	95.95
	Basal cell carcinoma	90.83	95.41	92.67	92.47
	Benign keratosis-like	84.12	76.33	80.03	79.90
	Dermatofibroma	99.05	98.67	99.56	98.50
	Melanocytic nevi	85.36	70.13	77.37	77.14
	Pyogenic granulomas and hemorrhage	99.45	98.07	99.05	99.50
	Melanoma	75.34	86.45	80.23	80.31
RF	Actinic keratoses	86.04	93.23	89.45	89.43
	Basal cell carcinoma	87.54	88.07	87.12	87.50
	Benign keratosis-like	75.87	72.14	74.35	73.48
	Dermatofibroma	95.78	99.56	97.76	96.98
	Melanocytic nevi	80.98	67.73	73.45	73.21
	Pyogenic granulomas and hemorrhage	97.09	99.13	98.56	97.99
	Melanoma	71.34	74.45	73.89	72.48
ANN	Actinic keratoses	93.12	99.43	96.08	95.95
	Basal cell carcinoma	95.34	96.45	93.78	95.50
	Benign keratosis-like	88.34	81.24	84.15	84.43
	Dermatofibroma	99.56	99.34	99.05	99.00
	Melanocytic nevi	84.67	75.15	79.08	79.37
	Pyogenic granulomas and hemorrhage	98.11	97.67	99.89	98.99
	Melanoma	82.34	89.19	85.45	85.43

**Fig. 9:**
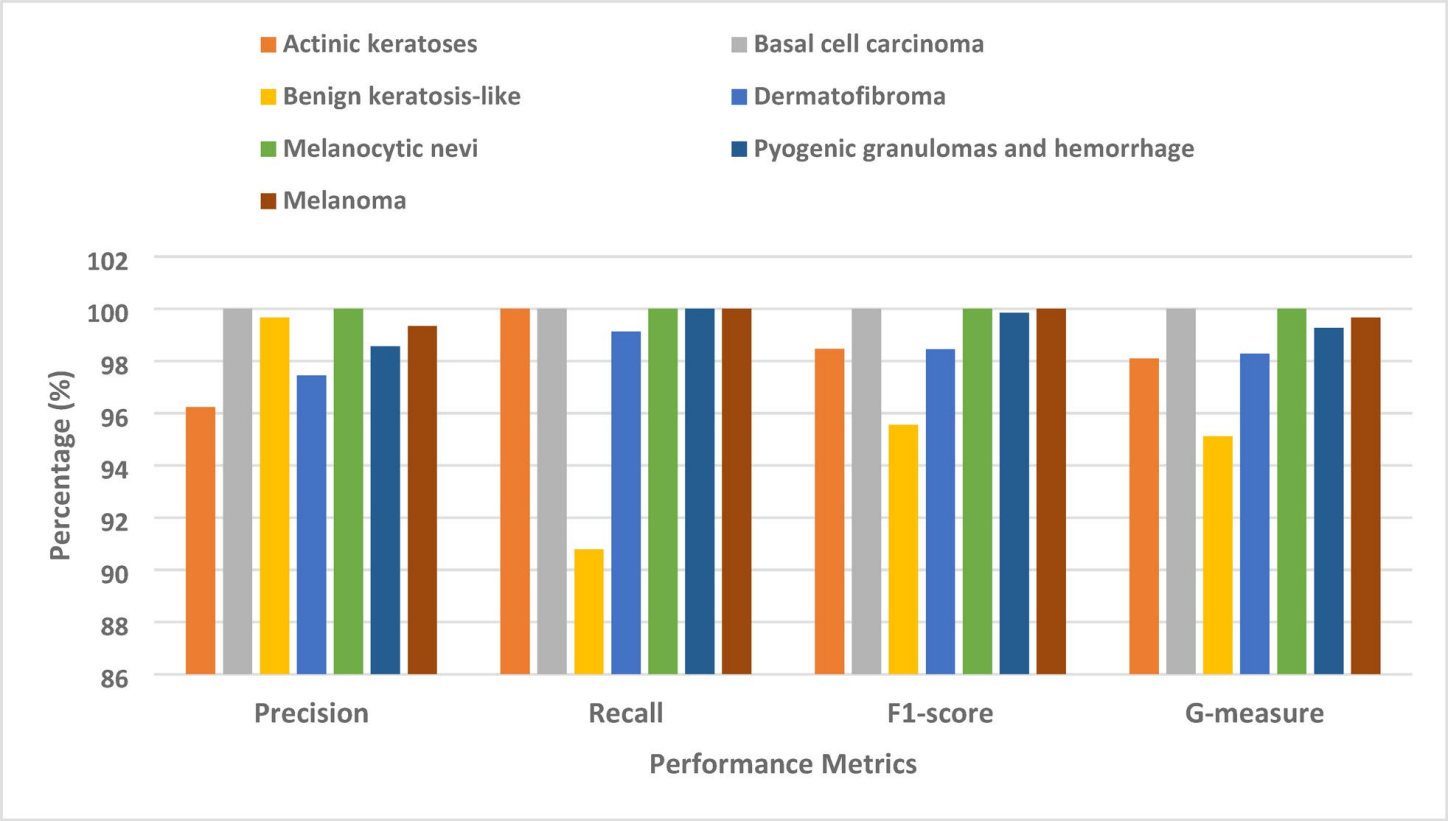
Result analysis of the proposed model.

Figure 8 provides a comparative performance analysis of six models namely ANN, RF, SVM, KNN, DT, and the proposed model based on four significant evaluation metrics: Precision, Recall, F1-score, and G-measure. The proposed model outperforms the traditional machine learning methods on all four metrics. The highest precision is achieved in the best model, which exceeds 90%, i.e., it produces fewer false positives and is more accurate in optimistic prediction than the other models. Similarly, the recall of the best model is also highest, i.e., it is better at identifying useful instances and minimizing false negatives compared to other classifiers. Since it is a harmonic mean of recall and precision, the proposed model again leads, guaranteeing that it better balances sensitivity and specificity compared to others. Notably, ANN and RF models exhibit similar performance in many measures; however, the model developed in this paper maintains a consistent lead in every category, particularly in terms of G-measure and precision. Traditional classifiers, such as DT and KNN, have relatively lower performance, suggesting limited applicability for tracking or classifying Melanoma and Non-Melanoma Skin Cancer. Figure 9 shows that the proposed model classifies the various types of skin lesions exceedingly well, with Precision, Recall, F1-score, and G-measure for all classes greater than 95%. The model shows particularly consistent and near-perfect precision for Melanocytic nevi, Basal cell carcinoma, and Melanoma. Despite small dips in performance in Benign keratosis-like lesions, Actinic keratoses, and Dermatofibroma, the overall result indicates a robust and consistent classification system with few mistakes.

### Comparative analysis of the confusion matrix for classifiers

**Fig. 10:**
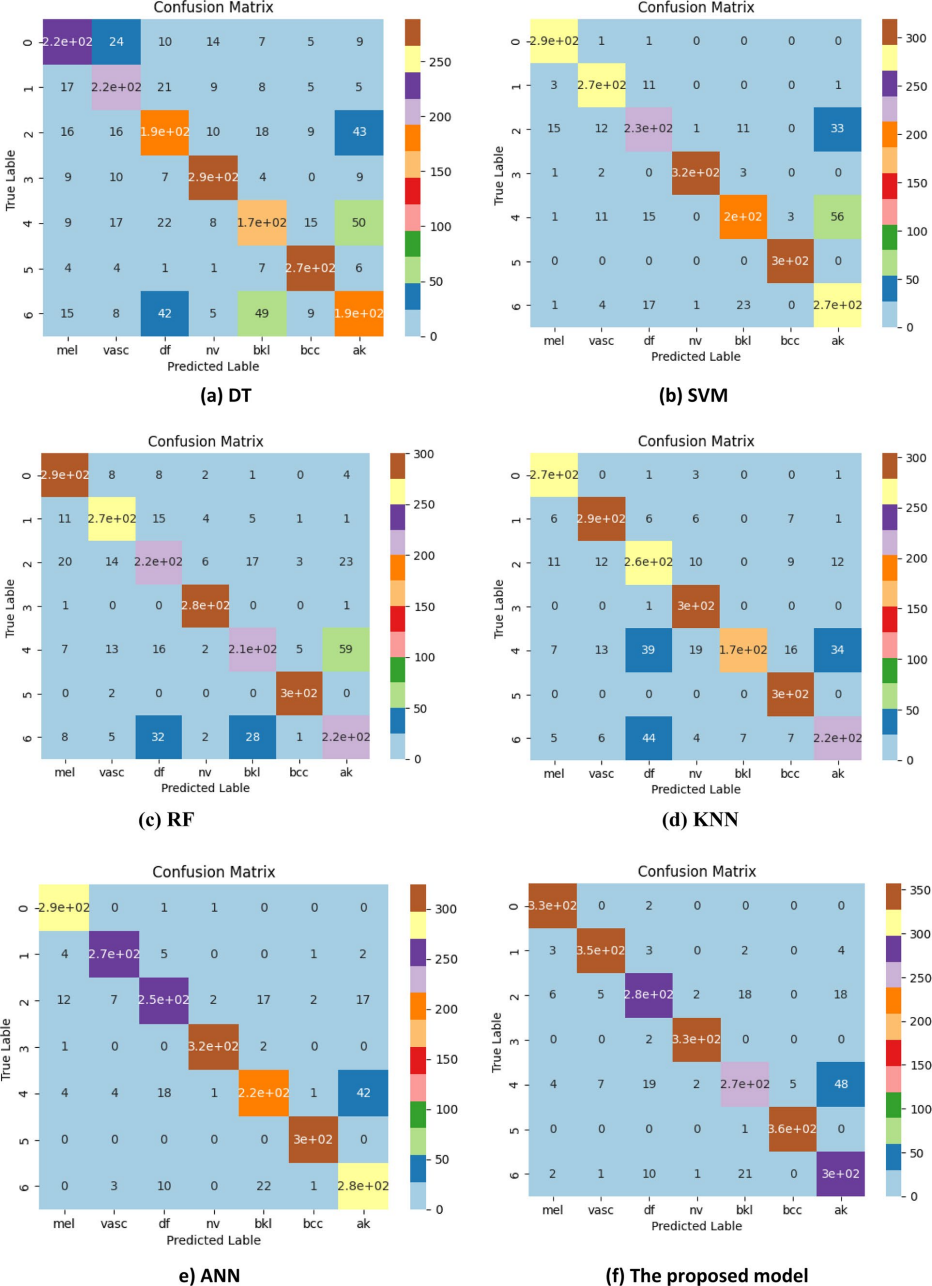
Confusion matrix for the DT, SVM, RF, KNN, ANN and the proposed model against the HAM10000 dataset.

The confusion matrices generated by the DT, SVM, RF, KNN, ANN, and the proposed model for skin lesion classification using the HAM10000 dataset are presented in Fig. 10. It showed that the proposed model had achieved an accuracy of 96.21%, as it predicted correctly a collection of 330 images of Melanoma, 350 images of Vascular lesions, 280 images of Dermatofibroma, 330 images of melanocytic nevi, 270 images of benign keratosis-like, 360 images to basal cell carcinoma and 300 images to actinic keratoses class. The obtained results demonstrate that the suggested model has successfully categorized the photos into separate class labels. Moreover, the designed model outperforms the competing models in terms of misclassifying photos. With a 91.78% success rate, the ANN model correctly identified 290 images as Melanoma, 270 as vascular lesions, 250 as Dermatofibroma, 320 as melanocytic nevi, 220 as benign keratosis-like lesions, 300 as basal cell carcinoma, and 280 as actinic keratoses. In contrast, the SVM model achieved an accuracy rate of 89.23%. It correctly identified 290 images as Melanoma, 270 images as vascular lesions, 230 images as Dermatofibroma, 320 images as melanocytic nevi, 220 images as benign keratosis-like, 300 images as basal cell carcinoma, and 270 images as actinic keratoses. Additionally, the KNN model achieves a very satisfactory result with an accuracy rate of 86.56%. The model correctly predicts that 270 images are Melanoma, 290 are vascular lesions, 260 are Dermatofibroma, 300 are melanocytic nevi, 170 are benign keratosis-like lesions, 300 are basal cell carcinoma, and 220 are actinic keratoses. Within the same framework, the RF model has attained an accuracy rate of 85.89%. It accurately predicted that 290 images were Melanoma, 270 images were vascular lesions, 220 images were Dermatofibroma, 280 images were melanocytic nevi, 210 images were benign keratosis-like, 300 images were basal cell carcinoma, and 220 images were actinic keratoses. Ultimately, the DT classifier achieved an accuracy rate of 73.67%. It correctly identified 270 images as basal cell carcinoma, 190 images as Dermatofibroma, 290 images as melanocytic nevi, 170 images as benign keratosis-like, and 190 images as actinic keratoses. Two hundred and twenty images were correctly identified as Melanoma.

### Comparison of the ROC curves of classifiers

**Fig. 11:**
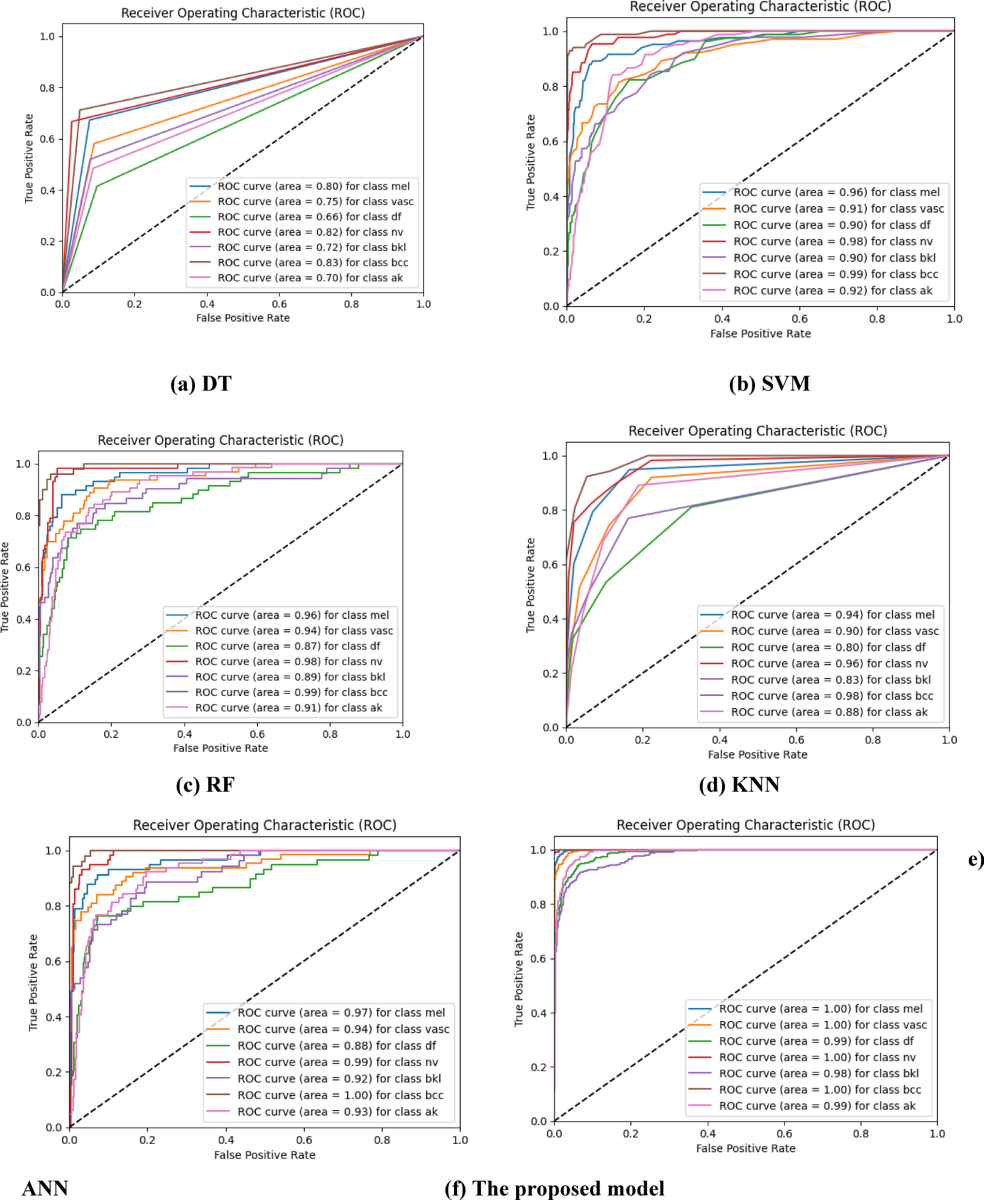
AUC analyses of ROC curves for (**a**) DT; (**b**) SVM; (**c**) RF; (**d**) KNN; (**e**) ANN; and (**f**) the proposed model against HAM10000 dataset.

We evaluate the efficiency of the framework in Fig. 11 using the area under the curve (AUC) curve and the receiver operating characteristic (ROC) curve. However, instead of relying solely on accuracy, which can be misleading due to precision constraints, the confusion matrix provides valuable information on both reliable and inaccurate categorizations at specific operational stages. This aids in mitigating accuracy limitations because its representation cannot completely capture the model’s performance across different operating conditions or when the model’s behavior changes. The receiver operating characteristic (ROC) graph illustrates the equilibrium between the rates of correctly identified positive cases and incorrectly identified false positive cases. The metric offers a more comprehensive evaluation of the model’s performance, extending beyond mere accuracy. The area beneath the receiver operating characteristic (ROC) curve, also referred to as the AUC value, is a metric that provides a meticulous assessment of the model’s performance by calculating its overall accuracy across various thresholds. The proposed model, along with the DT, SVM, RF, KNN, and ANN models, as well as the area under the curve (AUC) measurements of the HAM10000 dataset, are shown in Fig. 11. The proposed model has the greatest levels of accuracy in classifying skin lesions. For melanoma, vascular lesions, dermatofibroma, melanocytic nevi, benign keratosis-like, and basal cell carcinoma in the actinic keratosis group, the AUC values are 1, 1, 0.99, 1, 0.98, 1, and 0.99, in that order. Based on our analysis, the proposed model exhibits better performance compared to the fundamental classifiers. The artificial neural network (ANN) model achieves the highest area under the curve (AUC) score in two specific groups: “melanocytic nevi” and “basal cell carcinoma”. Both of these classes share identical attributes. By consistently maintaining AUC scores above 0.90 for all classes, it achieves the best overall performance. Conversely, the ‘basal cell carcinoma’ class has the highest score of 0.99 in the RF model, with the ‘melanocytic nevi’ category following closely at 0.98. Applying the DT model to the HAM10000 dataset is not advisable because of its significant features. This is primarily due to the DT classifier’s inability to provide a suitable match for the discrete features present in the data.

### Efficiency of the proposed approach

**Fig. 12:**
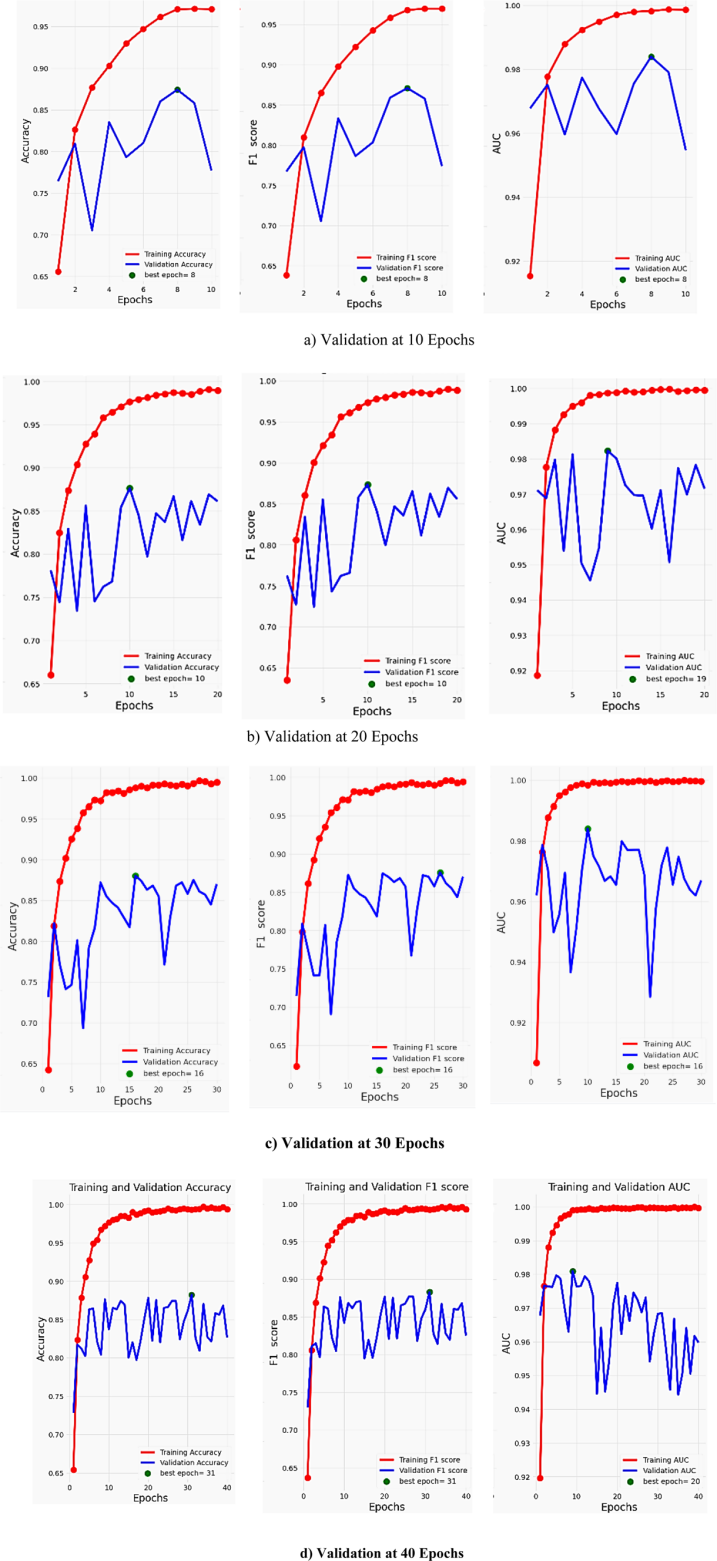
Performance of the proposed model throughout 10, 20, 30, and 40 Epochs in terms of accuracy, F1 score and AUC against HAM10000 dataset.

We assessed the efficiency of the proposed method by monitoring the F1 score, AUC, training, and validation accuracy over 10, 20, 30, and 40 epochs, as illustrated in Fig. 12. When the validation training exceeded the specified threshold of 0.99, the training procedure was terminated. At 8 epochs, the model achieved an optimal training accuracy of 0.97% and a validation accuracy of 0.87%. Moreover, Fig. 12a visually illustrates the convergence of the F1 score and AUC processes. We obtained weighted averages of accuracy, F1 score, and AUC parameters for evolution at 97%, 98%, and 99% for training and validation, respectively. Figure 12b illustrates the effectiveness of the model under consideration after 20 epochs. The validation accuracy of the suggested model initially stood at 66% but subsequently rose to 98%. The model presented in this study had similar F1 score values during both the training and validation stages, beginning at 0.64 and reaching a convergence point of 0.97. At the 40th epoch, the accuracy of the suggested model initially stood at 0.66 and gradually approached 1.0. Evaluation of the suggested model revealed that, when adjusted, it produces an unparalleled perceptual result in comparison to other conventional models. Nevertheless, the duration of the training and testing stages is a disadvantage of our methodology. This problem is rooted in the model’s structure. Therefore, our further efforts will involve streamlining the model in order to reduce the duration required for both training and testing.

Figure 12c illustrates the performance of the proposed model over 30 training epochs, tracking Accuracy, F1 Score, and AUC of the training and validation sets. The model’s performance on the training set, indicated by the red lines, keeps improving and saturates to near-perfect scores (~ 0.99–1.00) around epoch 20, indicating that the model is learning the training set. On the other hand, the validation metrics, which are blue lines, initially improve and then begin to oscillate as training continues wildly. The validation performance is highest at epoch 16, which is called the “best epoch”, but after that, the metrics show no systematic improvement and begin to get noisier. This divergence between the high, constant training metrics and the fluctuating validation metrics tells us that overfitting begins at epoch 16. Therefore, while the model possesses strong learning ability on the training set, the model’s capacity to generalize to novel data is compromised beyond epoch 16. Based on maximum validation Accuracy, F1 Score, and AUC, the best stop is at epoch 16, and applying early stopping at this epoch would most likely produce the best generalization performance on new data.

The training and validation performance of the proposed model after 40 epochs, as it is measured by Accuracy, F1 Score, and AUC, is illustrated in Fig. 12d. Red lines are utilized for training metrics, which continue to increase and plateau with scores very close to perfect (around 0.99–1.00), indicating that the model learned the training data well. However, the validation metrics, in blue, fluctuate wildly from epoch to epoch and do not have the smooth increasing trend of the training metrics. Validation Accuracy and F1 Score are best at epoch 31, and AUC is best at epoch 20. Beyond these points, the validation metrics display dramatic instability and do not keep increasing steadily, indicating the start of overfitting as the model continues training. The widening gap between training and validation curves confirms that the model’s generalization ability declines after these peak epochs. Thus, the best generalization performance happens at epoch 31 for Accuracy and F1 Score, and at epoch 20 for AUC. Applying early stopping at these respective epochs would likely provide the most reliable performance on unseen data.

### Results analysis using ISIC 2018 data set

The ISIC 2018 dataset, used extensively in this study, is a comprehensive resource for dermatological image analysis tasks such as lesion segmentation, classification, and cancer detection. Released as part of the ISIC challenge, this dataset supports research aimed at enhancing early detection strategies for skin cancer. It comprises 10,015 high-resolution dermoscopic images, each accompanied by expert-verified diagnostic labels. The dataset features a broad spectrum of lesion types, including BCC, SCC, and melanoma, captured across a variety of skin tones, lesion morphologies, sizes, and anatomical locations. This diversity ensures the development of machine learning models capable of generalizing well to real-world scenarios.

**Fig. 13:**
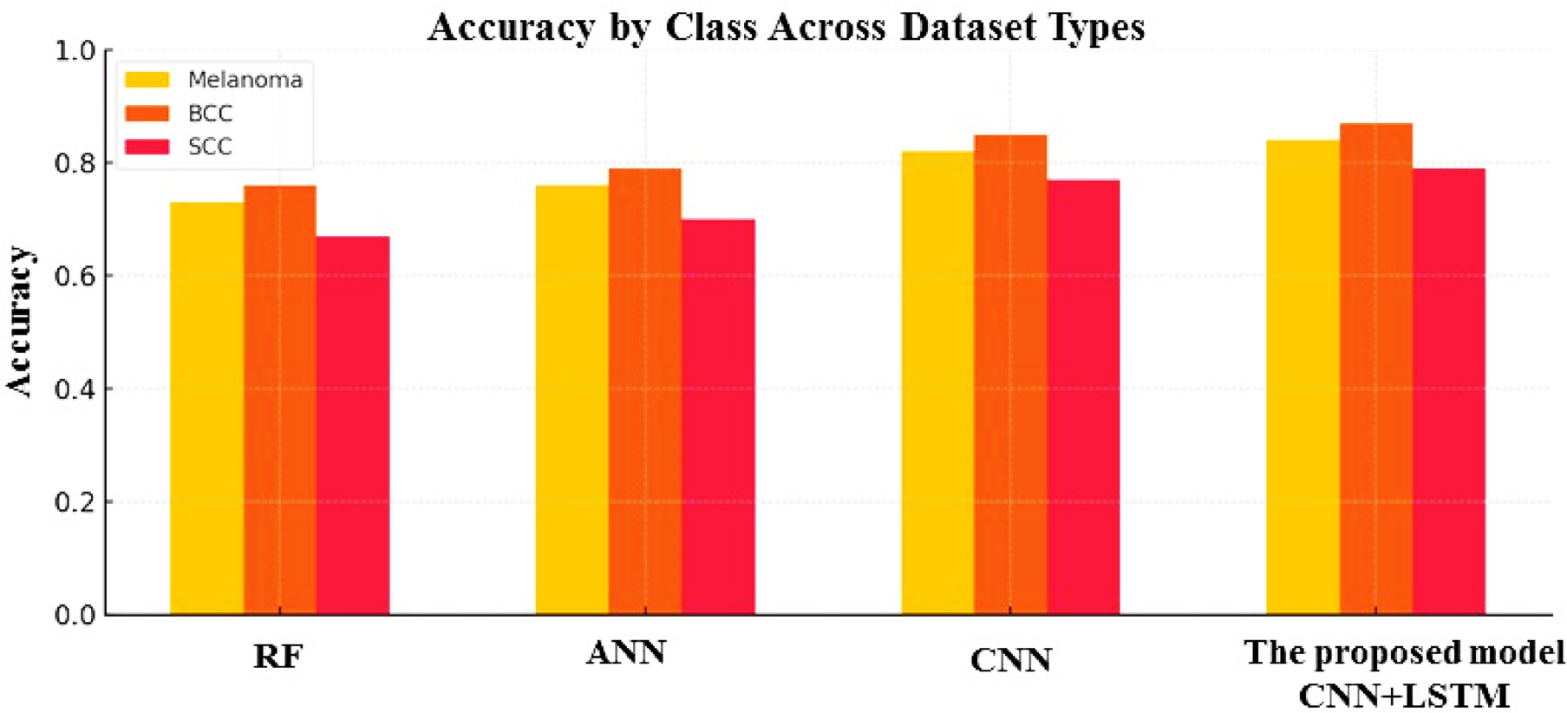
Comparative accuracy of skin lesion classification models across classes.

Figure 13 compares the accuracy of classification of four machine learning algorithms such as RF, ANN, CNN, and an idea CNN + LSTM model—on three types of skin lesions: Melanoma, BCC, and SCC. The proposed CNN + LSTM model consistently outperforms the other models across all classes, achieving the highest accuracy for Melanoma, BCC, and SCC. CNN also performs well, especially for BCC. ANN shows moderate performance, while RF has the lowest accuracy overall, particularly for SCC. This indicates that the integration of temporal features by means of LSTM with spatial features by means of CNN optimizes the performance of classification for skin lesion detection. The limitations of use LSTM in static image analysis frequently necessitate artificial sequences (such as patch-wise sequences), which may not provide appreciable performance gains and may cause the model to become overly complex. The computational cost, model size, and training time are all increased when CNNs and LSTMs are combined. When deploying in real-time or resource-constrained environments, such as embedded or mobile systems, this could be a drawback. LSTM layers add complexity to the already limited interpretability of CNNs, making it more difficult to debug and interpret the model, particularly in medical applications where explainability is essential.

### Performance analysis of the proposed model and various ML algorithms

The comparative analysis presented in Table 5 clearly shows that the projected CNN-LSTM patch-wise model outperforms several existing models on various datasets. The suggested approach yields well-balanced and efficient results, with an accuracy rate of 97%, precision of 92.23%, recall of 94.76%, and F1 score of 94.47%. The proposed approach outperforms several existing methods that either yield inferior results or provide incomplete analyses. While some, such as Moura et al.^19^ and Codella et al.^30^, reach accuracies of more than 93%, and others, such as Jaisakthi et al.^20^, reach strong F1-scores, none report complete sets of results for an end-to-end assessment. Additionally, a few of the models are data-specific or non-transferable to other data sources. In contrast, the proposed model, evaluated on the HAM10000 dataset, combines deep spatial feature learning with temporal learning, thereby improving its stable classification performance. In general, the suggested method provides a sound standard, with high accuracy and stable classification measures, indicating its potential for use in real-world autonomous skin lesion diagnostic systems.

**Table 5:** Comparison of proposed model with other existing works.

References	Datasets	Model	Accuracy%	Precision%	Recall%	F1-score%
Sanchez-Monedero et al.^10^	ISIC	CNN + SVM	91%			
Moura et al.^19^	PH2, DermIS	CNN	94.9%			
Jaisakthi et al.^20^	SIC-2019 ISIC-2020	DCNN	–			96.81
Jiang et al.^22^	ISIC	CNN-LSTM	87.01	86.75	89.43	87.12
Naqvi et al.^23^	ISIC	CNN-LSTM	93.41	–	–	–
Sai et al.^24^	HAM10000	CNN + RNN	94	–	–	–
Kumar et al.^29^	HAM10000	CNN + RNN	94%			
Codella et al.^30^	ISIC	CNN + SVM Sparse Coding,	93.1%	–	–	–
Taghreed et al.^35^	TCGA histopathology	CNN-LSTM	90	88	85	86
The proposed model	HAM10000	CNN-LSTM based patch wise	97%	92.23	94.76	94.47

## Conclusions and future works

In this paper, we proposed a novel hybrid deep learning architecture that combines LSTM networks with CNN to enhance the classification of melanoma and non-melanoma skin cancers using the HAM10000 dataset. By first employing LSTM layers, the model effectively captures temporal dependencies and sequential patterns in the input data, potentially modeling the spatial evolution of skin lesions. These temporal features are then passed to a CNN, which performs spatial analysis through time-distributed convolutional and pooling layers, extracting crucial visual characteristics such as texture, color, and edge information. The integration of LSTM and CNN enables the model to leverage both temporal and spatial features, allowing it to more effectively address the inherent complexity and variability of skin lesion images. Experimental evaluations demonstrate that the proposed LSTM-CNN hybrid model achieves superior performance compared to traditional single-architecture approaches, improving classification accuracy and reliability. Various evaluation metrics such as accuracy, recall, precision, F1 score, and ROC curve score are utilized to assess the model performance of the proposed LSTM-CNN model compared to conventional deep learning architectures. These results demonstrate the effectiveness and robustness of the hybrid approach in improving diagnostic accuracy.

Further research could explore several avenues to enhance the proposed model. Expanding the dataset to include more diverse and large-scale images may improve the model’s generalizability and robustness across different populations. Incorporating advanced deep learning techniques, such as attention mechanisms, could also enhance the model’s ability to focus on relevant regions of skin lesions, thereby increasing diagnostic accuracy. Additionally, integrating clinical data—such as patient history and genetic information—could contribute to developing a more comprehensive diagnostic tool. Finally, real-world validation through clinical trials is essential to confirm the model’s practical effectiveness in medical settings.

## Data Availability

HAM10000 dataset: https://api.isic-archive.com/collections/212/.

## Abbreviations

ANFIS: Adaptive neuro-fuzzy inference system
AK: Actinic keratosis
ANN: Artificial neural network
AUC: Area under the curve
BCC: Basal cell carcinoma
CAD: Computer-aided diagnosis
CRI: Radiograph image
CNN: Convolution neural networks
DF: Dermatofibroma
DT: Decision tree
FFNN: Feed-forward neural network
GLCM: Gray level co-occurrence matrix
GF: Gaussian filtering
HKNN: Hierarchical KNN classifier
ISIC: International Skin Imaging Collaboration
KNN: K-nearest neighbor
LBP: Local binary patterns
LSTMs: Long short-term memory networks
pyogenic gg: Pyogenic granulomas and hemorrhage
RF: Random forest
ReLU: Rectified linear unit
ROC: Receiver operating characteristic
RNN: Recurrent neural networks
SCC: Squamous cell carcinoma
SVM: Support vector machine
WHO: World Health Organization
ViTs: Vision transformers
XAI: Explainable AI
